# Economic cost of tobacco use in India, 2004

**DOI:** 10.1136/tc.2008.027466

**Published:** 2009-01-08

**Authors:** R M John, H-Y Sung, W Max

**Affiliations:** 1 University of Illinois at Chicago, Institute for Health Research and Policy, Chicago, Illinois, USA; 2 University of California San Francisco, San Francisco, California, USA

## Abstract

**Objective:**

To estimate the tobacco-attributable costs of diseases separately for smoked and smokeless tobacco use in India.

**Methods:**

The prevalence-based attributable-risk approach was used to estimate the economic cost of tobacco using healthcare expenditure data from the National Sample Survey, a nationally representative household sample survey conducted in India in 2004. Four major categories of tobacco-related disease—tuberculosis, respiratory diseases, cardiovascular diseases and neoplasms—were considered.

**Results:**

Direct medical costs of treating tobacco related diseases in India amounted to $907 million for smoked tobacco and $285 million for smokeless tobacco. The indirect morbidity costs of tobacco use, which includes the cost of caregivers and value of work loss due to illness, amounted to $398 million for smoked tobacco and $104 million for smokeless tobacco. The total economic cost of tobacco use amounted to $1.7 billion. Tuberculosis accounted for 18% of tobacco-related costs ($311 million) in India. Of the total cost of tobacco, 88% was attributed to men.

**Conclusions:**

The cost of tobacco use was many times more than the expenditures on tobacco control by the government of India and about 16% more than the total tax revenue from tobacco. The tobacco-attributable cost of tuberculosis was three times higher than the expenditure on tuberculosis control in India. The economic costs estimated here do not include the costs of premature mortality from tobacco use, which is known to comprise roughly 50% to 80% of the total economic cost of tobacco in many countries.

Knowledge of the health consequences of tobacco use has led to much greater reductions in tobacco use in developed than in developing countries. The tobacco epidemic is estimated to kill 8 million people annually, with 80% of deaths in developing countries by 2030.^1^ Smoking related medical costs account for 6% to 15% of healthcare costs in high-income countries.^2^ Evidence from developing countries such as China and Vietnam place this estimate around 4%.^3^ ^4^

With roughly 10% of the world’s smokers, India is the second largest consumer of tobacco in the world,^1^ second only to China. Tobacco consumption in India is characterised by a large proportion of non-cigarette and smokeless tobacco use. Manufactured cigarettes constitute only 14% of the tobacco consumption in India.^5^ The health effects of non-cigarette tobacco use are under-researched probably because they are not popular in most of the developed world. There is reason to believe that the non-cigarette tobacco used in India is also associated with significant adverse health outcomes. Bidis, an indigenous and popular smoked tobacco product in India, delivers nicotine, carbon monoxide and other toxic components of tobacco smoke in equal or greater amounts than conventional cigarettes,^6^ making bidi smoking a stronger risk factor than cigarette smoking for cancer of the hypopharynx and supraglottis.^7^ Many of the smokeless tobacco products in India such as Khaini, Mawa, Pan, Zarda and Gutkh are also found to be risk factors for cancer.^7^ Chewing tobacco in India is also a risk factor for oral cancers and esophageal cancers.^8^ A recent nationwide study on smoking and mortality in India estimated that cigarette and bidi smoking causes about 5% of all deaths in women and 20% of all deaths in men aged 30–69 years, totalling 1 million deaths per year in India in 2010.^9^

There has been no comprehensive national level study that estimated the economic cost of tobacco use in India. However, a report submitted to the government of India^10^ referred to a study by Rath and Chaudhry^11^ that estimated the cost of three major tobacco related diseases in India: cancer, coronary artery disease and chronic obstructive pulmonary disease. Based on a sample from 2 Indian locations—195 patients in Delhi and 500 patients in Chandigarh—they collected data on treatment expenditures (medical and non-medical), institutional expenditures and loss of wages during treatment for 1990–1992, or until death or recovery. Using the consumer price index, they estimated the total direct and indirect cost due to three major tobacco related diseases in India in 1999 to be Rs.277.61 ($6.2) billion, 83.7% of which was due to premature death. Reddy and Gupta^12^ updated these costs to 2002–2003, estimating the total cost for the three major tobacco related diseases to be Rs.308.33 ($6.6) billion.

Because tobacco use causes more than just the three diseases listed above, a more comprehensive estimate of the economic burden of tobacco use in India is needed. Moreover, the data needs to be recent and representative to the nation as a whole. This paper estimates the economic burden of tobacco use in India by considering four major categories of tobacco-related disease—tuberculosis, respiratory diseases, cardiovascular diseases and neoplasms—using nationally representative data. This provides the first ever estimate of the economic cost of tobacco in India using nationally representative data. This is also the first time economic costs are estimated separately for smoked and smokeless tobacco. Moreover, the tobacco-attributable cost of tuberculosis, a disease of major importance for India^13^ is estimated for the first time in India. Tobacco smoke is known to increase the risk of tuberculosis.^14^ ^15^ Recent epidemiological studies in India has also supported this claim.^9^

## METHODS

We use the prevalence-based attributable-risk approach applied to tobacco-related costs by Rice *et al*^16^ for estimating the economic burden of tobacco use. This approach measures the value of resources used (direct costs) or lost (indirect costs) from tobacco-caused diseases and deaths during a specified period of time, regardless of the time of tobacco use onset. This method of estimation is designed to measure the aggregate economic burden imposed on society attributable to tobacco use. Using a standard epidemiological formula, it determines the proportion of excess costs that can be attributed to tobacco use and hence preventable. We include only persons aged 35 years and older in the analysis, since relative risks for the diseases considered were available only for this age group.

### Data sources

The primary data source for estimating the tobacco-attributable medical cost was the “Morbidity, Health Care and the Condition of the Aged”,60th round of the National Sample Survey (NSS) conducted during January to June 2004. It was a nationally representative survey conducted by the National Ministry of Health and Family Welfare (MOHFW). It collected the data on utilisation and expenditures of private and public healthcare services—inpatient hospitalisation during the 365 days prior to the date of interview and outpatient visits during the 15 days prior to interview—from 47 302 rural and 26 566 urban households in India. Expenditures from inpatient hospitalisation were reported for each disease and visit separately. However, expenditures on outpatient visits were reported as total per person regardless of number of visits and ailments. In order to calculate the average expenditure per ailment per outpatient visit we computed average expenditures on outpatient visit for each ailment using only those patients with only one visit and that amount was imputed to the expenditures for the respective ailments of those with multiple visits and ailments. These 15-day averages were multiplied by 24.33 to get annual estimates. NSS self-reported household expenditures were scaled up by a factor of 1.68 to reflect the difference between NSS estimates and India’s national healthcare expenditures (expenditures by households, other private and public sources.^17^

The relative risk (RR) of mortality used to estimate the smoking attributable fraction was taken from a prospective 1992–1999 cohort study of 99 570 Mumbai adults aged 35 or older.^18^ They reported the RR, adjusted for age and socioeconomic status, separately for smokeless tobacco users and smokers by gender and disease categories. This is the only study that provides cause-specific RR separately for smoked and smokeless tobacco by gender, and thus is relevant in the Indian context where wide disparities in tobacco use exists between genders.^19^

The data source for estimating the prevalence of tobacco use was the second National Family Health Survey (NFHS-2)^20^ conducted by the International Institute for Population Sciences in 1998–1999. The NFHS-2 sample represented more than 99% of India’s population across all 26 states.

The population figure in India was taken from the estimated mid-year population for 2004 as projected by the US Census Bureau (http://www.census.gov/ipc/www/idb/tables.html), and was estimated to be 175 million males and 170 million females aged 35 and older.

### Estimation of the smoking attributable fraction

The smoking attributable fraction (SAF) is the proportion of expenditures on personal health services and morbidity costs that can be attributable to smoked and smokeless tobacco use. Persons were classified into three mutually exclusive categories: (1) never smokers: those who have never used any tobacco, (2) smokeless tobacco users: those who have used smokeless tobacco only and (3) smokers: those who have used smoked tobacco regardless of whether they also used smokeless tobacco or not (15% of adult males and 1% of adult females used smoked and smokeless tobacco). We estimated the SAF separately for smokeless tobacco users and smokers using an epidemiological formula derived from Lilienfeld and Lilienfield^21^ for each of the disease categories by gender and tobacco use type (equation 1).


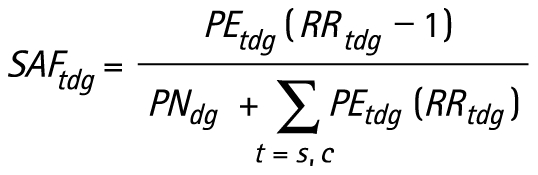


Subscripts t, d and g indicate type of tobacco users, disease category and gender, respectively. PN, PE_c_ and PE_s_ denote the percentage of people who are never smokers, smokeless tobacco users and smokers, respectively, with c and s denoting smokeless and smoked tobacco. RR_c_ and RR_s_ denote the relative risk of mortality for smokeless tobacco users and smokers, respectively, compared to never smokers.

### Estimation of economic cost

We considered three components of the economic cost of tobacco use: (1) direct healthcare expenditures for inpatient hospitalisation or outpatient visits, including surgeon’s fees, medicines, diagnostic tests, bed charges, attendant charges, medical appliances and ambulatory services; (2) expenditures incurred for transportation other than ambulance and lodging charge of caregivers; and (3) wage income lost to the whole household due to inpatient hospitalisation or outpatient visits. Items 2 and 3 comprise indirect morbidity costs. A fourth and important component of the economic cost of tobacco is the cost of premature deaths from tobacco use. Data were not available to estimate this component of cost.

Tobacco-attributable direct healthcare expenditures (TAE) are the product of total healthcare expenditure (THE) and the SAF:

TAE_tdg_ = THE_tdg_ × SAF_tdg_ = [PH_tdg_ × QH_tdg_ + PV_tdg_ × QV_tdg_ × 24.33] × POP_g_ × SAF_tdg_

Where PH is the average expenditure per inpatient hospitalisation, QH is the average number of hospitalisations per person in 365 days, PV is the average expenditure per outpatient visit and QV is the average number of outpatient visits per persons for 15 days prior to the date of interview. POP is the population aged > = 35 in 2004.

The tobacco-attributable indirect morbidity cost (TAI) is the product of total indirect morbidity expenditure (TIE) and the SAF:

TAI_tdg_ = TIE_tdg_ × SAF_tdg_ = [(PHI_tdg_ + PHL_tdg_) × QH_tdg_ + (PVI_tdg_ + PVL_tdg_) × QV_tdg_ × 24.33] × POP_g_ × SAF_tdg_

Where PHI and PVI are the average expenditure on transportation and caregivers per inpatient hospitalisation and per outpatient visit, respectively; PHL and PVL are the average income lost due to absence from work from inpatient hospitalisation and outpatient visits.

## RESULTS

### SAFs for smoked and smokeless tobacco

Table 1 shows the prevalence of tobacco use and the relative risks of mortality used in this paper. The prevalence of ever smokers in the age group 35+ for 1998–1999 was 42.3% and 4.4% for males and females while the prevalence of ever smokeless tobacco users was 20.7% and 18.8% for males and females. Prevalence of smoking is less among women in India due to social unacceptability^12^ and is largely a cultural phenomenon. The relative risks of mortality were slightly higher for smokers than smokeless tobacco users but did not differ much by gender or disease category, with the exception of tuberculosis, which was especially high for female smokers.

**Table 1:** Prevalence of tobacco use and disease-specific relative risk of mortality from tobacco use in India by type of tobacco users and gender for adults aged 35 and older

	Smoked		Smokeless	
	Male	Female	Male	Female
Prevalence* (%)	42.3	4.39	20.65	18.84
Relative risk of death:				
Respiratory diseases	2.12	1.15	1.50	1.04
Tuberculosis	2.30	5.92	1.46	1.40
Cardiovascular diseases	1.54	1.46	1.32	1.15
Neoplasm	2.60	1.85	1.40	1.57

*The prevalence of smokeless tobacco users includes those who only used smokeless tobacco whereas prevalence for smokers includes some who also used smokeless tobacco so that adding smoked and smokeless tobacco user would give prevalence for any tobacco use.

Table 2 shows the SAF of tobacco use by type of tobacco user, gender and disease category computed based on the data shown in table 1 and equation 1. For smokers, the SAFs are substantially lower for women than men for all disease categories partly due to their low smoking prevalence. Cancer and tuberculosis show the highest and the second highest disease-specific SAFs respectively for men, while tuberculosis has the highest disease-specific SAF for women. As for smokeless tobacco users, the SAFs between men and women are not much different. Combining smoked and smokeless tobacco use, the SAFs among men range from 22.8% for cardiovascular diseases to 43.2% for neoplasms. Considering that the SAFs among women are up to 22.6% for tuberculosis and 12.6% for neoplasms, tobacco use contributes to a significant proportion of the burden on Indian women’s health despite the low prevalence of tobacco use among them.

**Table 2:** Disease-specific smoking attributable fractions (SAFs) (%) in India by type of tobacco use and gender for adults aged 35 and older

Cause of death	Smoked		Smokeless		All tobacco*	
	Male	Female	Male	Female	Male	Female
Respiratory diseases	30.04	0.65	6.55	0.74	36.59	1.39
Tuberculosis	33.43	16.73	5.77	5.84	39.21	22.56
Cardiovascular diseases	17.65	1.93	5.10	2.70	22.75	4.62
Neoplasm	38.47	3.26	4.69	9.38	43.16	12.64

*The SAF for all tobacco products equals the SAF for smokers plus the SAF for smokeless tobacco users.

### The economic costs of tobacco use

Table 3 shows the economic cost of tobacco use for India in 2004 by disease, type of tobacco use and gender separately for inpatient hospitalisation and outpatient visits. The top section of table 3 shows TAEs. The total TAE of treating tobacco related diseases amounted to $1192.5 million, including $833.9 million for male smokers, $73.2 million for female smokers, $188.7 million for male smokeless tobacco users and $96.6 million female smokeless tobacco users. The TAEs were greater for males than females for all disaggregated analyses except that females have higher TAEs for treating cancer attributable to smokeless tobacco use. The TAEs were highest for cardiovascular diseases for males and females regardless of the type of tobacco use. The total TAE from smoked tobacco ($907.1 million) was more than three times that from smokeless tobacco ($285.3 million).

**Table 3:** Economic costs of tobacco use in India for 2004 among adults aged 35 and older (US $1000)

Disease	Smoked					Smokeless					All tobacco		
	Inpatient		Outpatient		Subtotal	Inpatient		Outpatient		Subtotal	Male	Female	Total
	Male	Female	Male	Female		Male	Female	Male	Female				
	Tobacco-attributable direct healthcare expenditure												
Respiratory	27 994	548	264 032	3533	296 107	6101	627	57 542	4044	68 314	355 669	8 752	364 421
Tuberculosis	34 604	5442	103 035	18 076	161 157	5978	1899	17 798	6307	31 981	161 415	31 723	193 139
Cardiovascular	102 003	7241	208 625	24 859	342 728	29 508	10 133	60 354	34 788	134 783	400 490	77 021	477 511
Neoplasm	41 380	8317	52 276	5176	107 149	5050	23 936	6380	14 897	50 263	105 086	52 327	157 412
Subtotal	205 980	21 548	627 968	51 644	907 141	46 637	36 595	142 074	60 035	285 342	1 022 660	169 823	1 192 482
	Tobacco-attributable transportation and caregivers expenditure												
Respiratory	1521	29	18 600	346	20 497	332	34	4054	396	4815	24 506	806	25 312
Tuberculosis	1585	239	10 383	1439	13 646	274	84	1793	502	2653	14 035	2265	16 299
Cardiovascular	3598	221	17 954	2188	23 962	1041	310	5194	3062	9607	27 787	5781	33 568
Neoplasm	2733	363	8343	577	12 016	334	1044	1018	1662	4057	12 428	3646	16 074
Subtotal	9437	853	55 279	4551	70 121	1980	1471	12 059	5622	21 132	78 756	12 497	91 253
	Tobacco-attributable lost income due to absence from work												
Respiratory	2061	41	115 432	614	118 148	449	47	25 157	703	26 356	143 099	1405	144 503
Tuberculosis	3337	493	74 019	7670	85 519	576	172	12 786	2676	16 211	90 718	11 012	101 730
Cardiovascular	3936	216	81 334	2913	88 399	1139	303	23 529	4076	29 047	109 938	7508	117 446
Neoplasm	3274	381	30 452	2153	36 261	400	1098	3716	6197	11 411	37 842	9830	47 672
Subtotal	12 608	1132	301 237	13 351	328 327	2564	1619	65 189	13 653	83 025	381 597	29 755	411 352
Total	228 025	23 533	984 484	69 546	1 305 589	51 181	39 686	219 322	79 310	389 499	1 483 013	212 075	1 695 087

The middle and lower sections of table 3 show the tobacco-attributable indirect morbidity costs for 2004. The total tobacco-attributable transportation and caregiver costs amounted to $91.3 million, including $64.7 million for male smokers, $5.4 million for female smokers, $14.0 million for male smokeless tobacco users and $7.1 million for female smokeless tobacco users. Cardiovascular disease accounts for the largest share of transportation and caregiver costs for males and females. The total value of lost income from tobacco related hospitalisation and outpatient visits amounted to $411.4 million, including $313.8 million for male smokers, $14.5 million for female smokers, $67.8 million for male smokeless tobacco users and $15.3 million for female smokeless tobacco users.

The last row of table 3 presents the total economic cost. Of the total cost of $1.7 billion, smoked tobacco accounts for 77% vs 23% for smokeless tobacco; 87% is attributed to males vs 13% to females. Females contribute more to the cost of smokeless tobacco (31%) than to smoked tobacco (7%). This is reflective of the fact that the prevalence of smokeless tobacco use among women is 19% compared to only 4% for smoked tobacco use.

## DISCUSSION

This paper presents the first comprehensive estimate of the economic burden of tobacco use at the national level for India. The total economic cost of tobacco use in India for 2004 amounted to $1.7 billion, which is many times more than the $551 876 that the government of India spent on tobacco control activities in 2006,^1^ and is 16% more than the total excise tax revenues collected from all tobacco products in India in the financial year 2003–2004 ($1.46 billion). Tobacco-attributable direct costs ($1.2 billion) account for 4.7% of India’s total national healthcare expenditure in 2004 ($25 billion).^17^ In comparison, studies from other developing countries such as China^4^ and Vietnam^3^ found the direct cost of smoking to be 3.1% and 4.3% of the national healthcare expenditure, respectively.

Tuberculosis is a major health risk in India with roughly 1.8 million new cases reported annually,^13^ and our findings highlight the important role of tobacco use for this disease. In fact, tuberculosis accounts for $311 million (18%) of the total economic cost of tobacco use in India, including $193 million (16%) of the direct cost and $118 million (24%) of the indirect morbidity cost. This is more than three times the $100 million that was spent on tuberculosis control in India in the year 2006.^13^

One limitation of our study is that we used relative risk of mortality to estimate the attributable morbidity. Risks of morbidity and mortality from tobacco use need not be the same. However, this approach has been widely used in the literature.^4^ ^22^^–^24 Two other approaches have been used to estimate the SAFs for direct medical cost. One was originally developed by Rice and Hodgson (1986),^16^ in which the RR of healthcare utilisation for smokers was first estimated and then applied to the calculation of the SAFs for medical cost. The other one was developed in the 1990s by several health economists,^25^^–^27 in which the SAFs was estimated directly from multiple-equation econometric models of the impact of smoking on healthcare expenditures. Due to our data limitations, we could not employ either approach in this study. However, according to a study by Rice and Hodgson,^16^ the SAFs for direct medical cost estimated by using the RR of healthcare utilisation approach was 23.5%, while the SAFs for direct medical cost of smoking estimated by using the RR of mortality approach was 19.7%. Therefore, the use of RR of mortality as a proxy for RR of healthcare utilisation is expected to yield an underestimated and conservative SAF for medical costs. Secondly, the relative risks we used were taken from a cohort study of 99 570 persons in Mumbai that is not nationally representative. Longitudinal data on risk factors for healthcare expenditures would be required to apply econometric models to cost estimation. With these data, one could control for different risks, assess the source of payments specifically for tobacco-related diseases and consider the impact of cessation on healthcare expenditure. Unfortunately these data are not available for India.

**What this paper adds**

- The economic cost of tobacco use has been estimated in many countries. However, to date there has not been any comprehensive national level study that estimated the economic cost of tobacco use in India in spite of the fact that India is the second largest consumer of tobacco in the world.
- This study estimates that the economic cost of tobacco use in India, not including the premature mortality cost, amount to $1.7 billion in the year 2004. Of this, smoked tobacco accounted for 77% and smokeless tobacco 23%; 87% is attributed to males and 13% to females.

Our estimates are probably low. We were unable to include the costs of premature mortality from tobacco use, because data on number of deaths by underlying cause of death at the national level in India are currently difficult to acquire. However, the estimates presented here are still important because it is the first time economic costs of tobacco use in India are presented using nationally representative healthcare expenditure data. Even the conservative estimates presented here are huge in comparison with the taxes collected from tobacco or the expenditure on tobacco control incurred by Government of India. The mortality cost has been estimated to account for 84% of total tobacco-related costs in India.^11^ Studies from China,^4^ Korea,^28^ USA,^29^ and Germany^30^ estimate the cost of premature death to be 58%, 91%, 46% and 64% of the total cost of smoking respectively. If the value of tobacco-attributable deaths adds 84% to the total costs, our estimate of the total economic costs of tobacco use in India for 2004 would be $10.6 billion. It should be also noted that due to the general assumptions used for earnings and employment, the indirect costs especially for women might be under-estimated. Furthermore, our analysis is limited to four categories of tobacco-caused disease. Many more diseases are known to be caused or exacerbated by tobacco use.

The huge healthcare burden attributable to tobacco use in India has many dimensions. More than 70% of the healthcare cost in India is out-of-pocket expenditures. Given that consumption of tobacco in India is more prevalent among the poor,^31^ it is likely that much of the tobacco related illness and the associated economic cost would also be higher among them. Hospitalisations for tobacco related diseases force poor people into debt traps and can result in severe impoverishment. There is a higher risk (odds ratio 1.35) of borrowing and distress selling during hospitalisation by individuals who use tobacco in India.^32^ Expenditures on tobacco in India displace expenditures on food and education.^33^ Thus, high spending on tobacco coupled with the higher healthcare burden of treating tobacco related diseases can push tobacco consumers into a vicious circle of tobacco use, ill health and poverty.

The economic cost of tobacco use in India reflects an important gender dimension, with 87% costs accounted for by males. Yet, the consequent toll on household income is shared by all the household members. Tobacco control efforts should take heed of these different dimensions of the economic costs of tobacco use.

Current economic costs associated with tobacco use are much higher than the tax revenues generated from tobacco. There is also evidence that the taxes on tobacco in India are much lower than the optimum level possible.^34^ Hence, an increase in tobacco taxes could be justified and that money could be used to pay for tobacco induced healthcare expenditures for the poor and for tobacco control efforts to prevent these diseases and lower these costs. An increase in tobacco taxes can also reduce expenditures on tobacco as increased taxes are known to result in decreased tobacco use.^35^
